# Crystal structure of ethyl 4-[(4-methyl­benz­yl)­oxy]benzoate

**DOI:** 10.1107/S2056989022009380

**Published:** 2022-09-30

**Authors:** Md. Hasan Al Banna, Md. Rezaul Haque Ansary, Ryuta Miyatake, Md. Chanmiya Sheikh, Ennio Zangrando

**Affiliations:** a Department of Chemistry, Rajshahi University, Rajshahi-6205, Bangladesh; b Center for Environmental Conservation and Research Safety, University of Toyama, 3190 Gofuku, Toyama, 930-8555, Japan; c Department of Applied Science, Faculty of Science, Okayama University of Science, Japan; d Department of Chemical and Pharmaceutical Sciences, University of Trieste, Italy; Vienna University of Technology, Austria

**Keywords:** crystal structure, ester, ether, conformational flexibility

## Abstract

The title compound, C_17_H_18_O_3_, crystallizes with three mol­ecules in the asymmetric unit. The mol­ecules differ in the conformation related to the eth­oxy group and in the orientation of the two phenyl rings, one of which has the eth­oxy group disordered over two positions with refined occupancies of 0.735:0.265 (9). In the crystal packing, the mol­ecules are connected by weak C—H⋯π inter­actions.

## Abstract 2

Three mol­ecules of the title compound are present in the asymmetric unit, exhibiting different conformations relative to the eth­oxy group.

## 1. Chemical context

Alkyl­benzoates are an important class of compounds with inter­esting physical properties and applications in industry. For example, 4-hy­droxy­benzoic acid and its esters are widely used as preservatives in cosmetic and pharmaceutical products known as parabens, for which the physical properties and crystal structures have been widely described (Giordano *et al.*, 1999 ▸; Yang *et al.*, 2014 ▸).

Alkyl­benzoates of different properties have been designed, amongst other things, with the aim of preparing liquid crystalline compounds (Abser *et al.*, 1993 ▸), functionalized poly(benzyl ether) dendrimers with methyl ester decorations as efficient organogelators (Feng *et al.*, 2009 ▸), or non-linear optical materials (Perumal *et al.*, 2002 ▸). Moreover, the ester bond has a prominent position in cell biology and medicinal chemistry (Lavis, 2008 ▸), and carbohydrazones can be obtained by reacting corresponding esters with suitable hydrazine derivatives. 
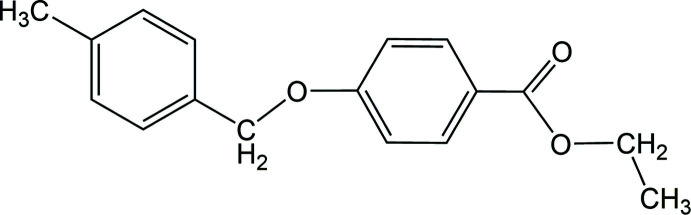


We report here the synthesis and crystal structure of another example of a derivatized alkyl­benzoate with an ether group.

## 2. Structural commentary

**Figure 1:**
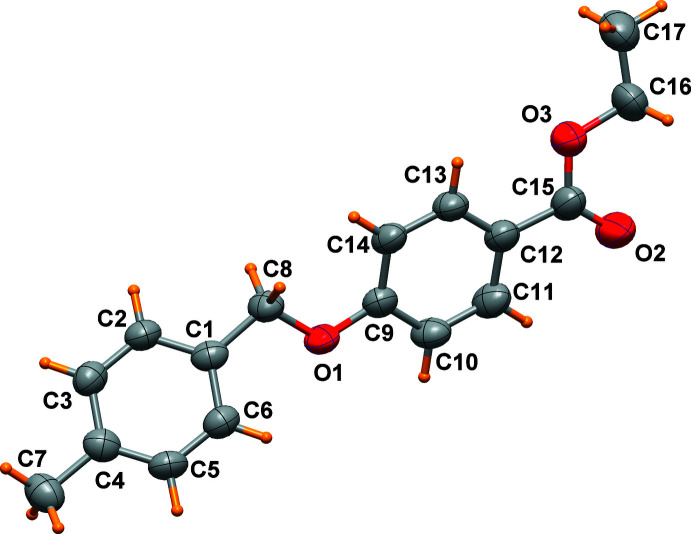
Mol­ecule *A* of the title compound, drawn with displacement ellipsoids at the 50% probability level.

**Figure 3:**
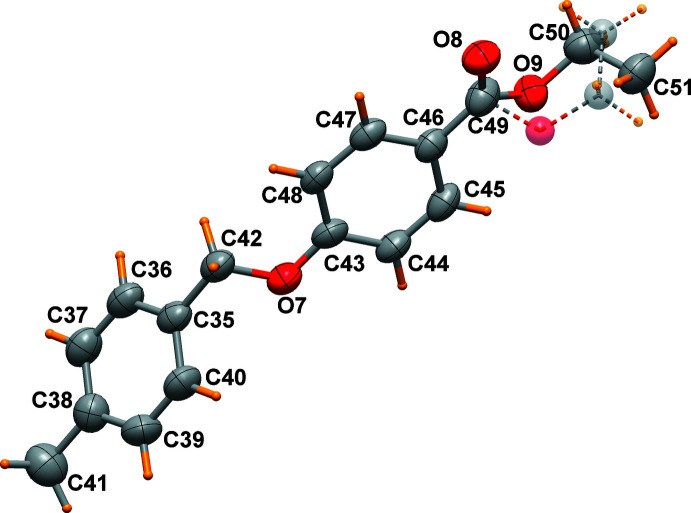
Mol­ecule *C* of the title compound, drawn with displacement ellipsoids at the 50% probability level. The eth­oxy group O9/C5/C51 is disordered over two sets of sites.

**Figure 4:**
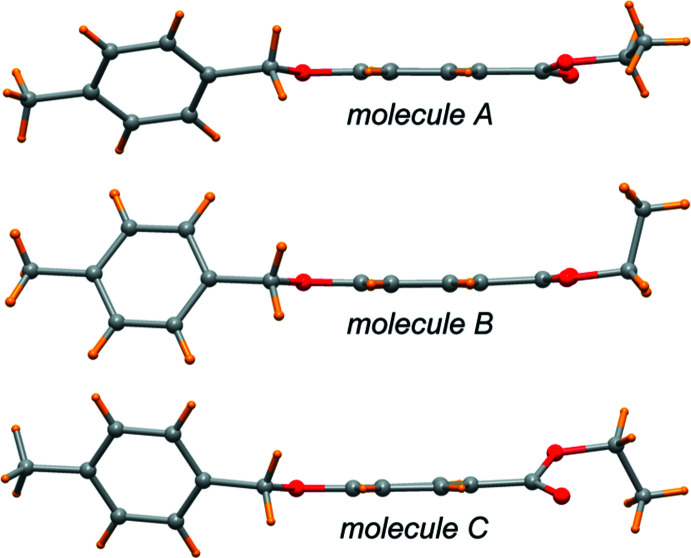
Side view of the three independent mol­ecules displaying the different conformations.

Three mol­ecules, which slightly differ in their conformations, are present in the asymmetric unit of the title compound (Figs. 1 ▸–3 ▸ ▸). The main conformational differences of mol­ecules *A*, *B* and *C* are related to the eth­oxy group with C—O—CH_2_—CH_3_ torsion angles of 174.0 (6), 82.6 (6) and 89.6 (7)°, and in the orientation of the two phenyl rings that form a dihedral angle of 46.4 (1), 70.3 (1), and 62.2 (1)°, respectively. A side view of the mol­ecules displayed in Fig. 4 ▸ highlights these differences. All these features are indicative of the conformational freedom of this mol­ecule. Nevertheless, all bond lengths and angles in the three mol­ecules relating to the ether and the ester groups are similar within their standard uncertainties. In general, bond lengths (Allen *et al.*, 1987 ▸) and angles are within normal ranges. In mol­ecule *C*, the eth­oxy group O9/C5/C51 is disordered over two sets of sites (Fig. 3 ▸).

In the parent methyl 4-(benz­yloxy)-3-meth­oxy­benzoate compound, which is an important organic inter­mediate for the synthesis of the anti­neoplastic drug Cediranib (Wang *et al.*, 2013 ▸), the two aromatic rings are almost normal to each other forming a dihedral angle of 85.81 (10)° and bond lengths are close comparable with those determined here.

## 3. Supra­molecular features

**Figure 5:**
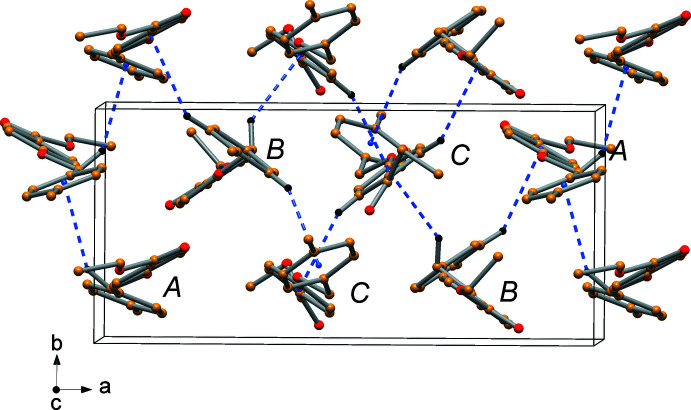
Perspective view of the crystal packing of the title compound down the *c* axis with indication of the C—H⋯π-ring inter­actions. H atoms not involved in the inter­actions were omitted for clarity.

Despite the number of phenyl rings, the aromatic rings have rather distant centroid-to-centroid distances of between 4.727 (3) and 4.946 (3) Å, but with unsuitable orientations for efficient π-stacking inter­actions. On the other hand, the crystal packing indicates a series of C—H⋯π ring inter­actions in the range 2.65–2.94 Å (Table 1 ▸), as derived with *PLATON* (Spek, 2020 ▸). A view of the unit cell is displayed in Fig. 5 ▸, showing these kinds of inter­actions. In addition, non-conventional C—H⋯O hydrogen bonds are observed in the crystal packing (Table 2 ▸).

## 4. Database survey

The conformations of the three independent mol­ecules present in the crystal structure of the title compound agree with previous structurally characterized species containing the (benz­yloxy)phenyl fragment, where the two aromatic rings form dihedral angles of 64.5 (2)° (mean value of two independent mol­ecules; Bats & Canenbley, 1984 ▸) and 69.19 (6)° (Qin *et al.*, 2019 ▸). However, a few structures exhibit almost coplanar orientations of the phenyl rings (Jasinski *et al.*, 2008 ▸; Feng *et al.*, 2009 ▸), or small dihedral angles such as the 4.1 (2) and 10.9 (4)° reported for 3,5-bis­(benz­yloxy)benzoic acid (Moreno-Fuquen *et al.* 2012 ▸). The latter conformations favour electron delocalization between the two rings, but packing requirements also play a role.

## 5. Synthesis and crystallization

A mixture of ethyl-4-hy­droxy­benzoate (8.75 g, 52.65 mmol) and 4-methyl­benzyl­bromide (9.75 g, 52.68 mmol) in acetone (100 ml) was refluxed for 14 h over anhydrous potassium carbonate (20 g). The solvent was removed *in vacuo*, and the remaining solid was dissolved in water and extracted with di­chloro­methane. Left overnight, colourless needle-shaped crystals were formed, filtered off, washed, and dried over silica gel in a desiccator. Yield: 12.58 g, 88% Melting point: 323 −324 K. FT–IR: 1706 ν (C=O), 1258, 1276 ν (C—O_ester_), 1106, 1102 ν (C—O_ether_). ^1^H NMR (CDCl_3_,600 MHz): δ = 1.37 (*t*, 3H, CH_3_CH_2_-, *J* = 10.5 Hz), 2.36 (*s*, 3H, C_6_H_4_–CH_3_),4.35 (*q*, 2H, CH_3_–CH_2_, *J* = 10.5 Hz), 5.07 (*s*, 2H, C_6_H_4_–CH_2_–), 6.98 (*d*, 2H, H-5,6, *J* =7.8 Hz), 7.20 (*d*, 2H, H-10,11, *J* = 11.4 Hz), 7.31 (*d*, 2H, H-8,9, *J* = 12 Hz), 7.99 (*d*, 2H, H-3,4, J = 6.6 Hz), ppm. ^13^C NMR (CDCl_3_, 600 MHz): 14.4 (C11), 21.3 (C7), 60.7 (C10), 70.1 (C8), 114.4 (C-3,5), 123.15 (C1), 127.73 (C-2′,6′), 129.1 (C-3′,5′), 131.6 (C-2,6), 133.31 (C1′),138.13 (C4), 162.5 (C4′), 166.4 (C9), ppm. LC–MS (ESI) *m*/*z*: [*M* + H]^+^. Calculated for C_17_H_18_O_3_ 271.13; found 271.13.

## 6. Refinement

Crystal data, data collection and structure refinement details are summarized in Table 3 ▸. The structure was refined as a two-component inversion twin. The –OCH_2_CH_3_ moiety of mol­ecule *C* was found to be disordered over two sets of sites with refined occupancies of 0.735 (9):0.265 (9). For modelling the minor disordered part, all atoms were refined with isotropic displacement parameters, and C—C and C—O bond lengths were restrained by using DFIX commands.

## Supplementary Material

Crystal structure: contains datablock(s) I. DOI: 10.1107/S2056989022009380/wm5660sup1.cif

Structure factors: contains datablock(s) I. DOI: 10.1107/S2056989022009380/wm5660Isup2.hkl

Supporting information file. DOI: 10.1107/S2056989022009380/wm5660Isup3.cml

CCDC reference: 2174691

Additional supporting information:  crystallographic information; 3D view; checkCIF report

## Figures and Tables

**Figure 2:**
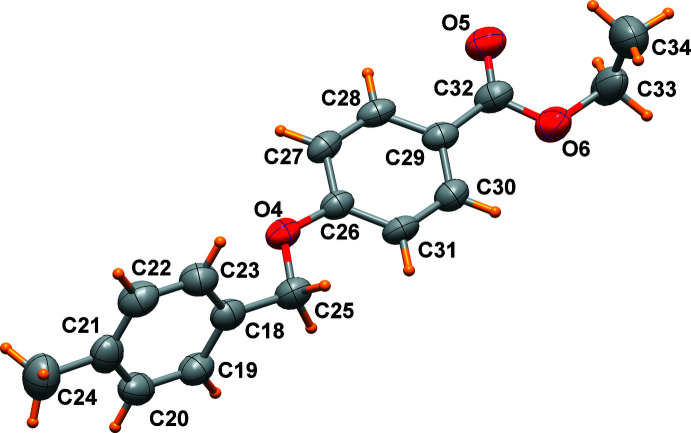
Mol­ecule *B* of the title compound, drawn with displacement ellipsoids at the 50% probability level.

**Table 1:** Analysis of C—H⋯*Cg*(π-ring) inter­actions (Å, °) C—H⋯π = angle of the *X*—H bond with the π-plane (perpendicular = 90°, parallel = 0°). Ring *Cg*1 = C35–C40; *Cg*2 = C43–C48; *Cg*3 = C1–C6; *Cg*4 = C9–C14; *Cg*6 = C26–C31

C—H	*Cg*(*J*)	Symmetry code (*J*)	H⋯*Cg*	C—H⋯*Cg*	C⋯*Cg*	C—H⋯π
C8—H8*A*	*Cg*3	-*x*, 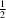 + *y*, −*z*	2.90	144	3.749 (5)	59
C20—H20	*Cg*1	*x*, *y*, *z*	2.81	147	3.653 (6)	62
C22—H22	*Cg*4	*x*, 1 + *y*, 1 + *z*	2.93	137	3.688 (6)	50
C25—H25*A*	*Cg*2	*x*, 1 + *y*, *z*	2.81	143	3.654 (6)	50
C41—H41*B*	*Cg*1	1 − *x*, 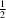 + *y*, 3 − *z*	2.67	156	3.590 (5)	75
C45—H45	*Cg*6	*x*, *y*, *z*	2.82	147	3.654 (4)	51
C47—H47	*Cg*2	1 − *x*, − 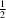 + *y*, 2 − *z*	2.77	148	3.615 (4)	65

**Table 2:** Hydrogen-bond geometry (Å, °)

*D*—H⋯*A*	*D*—H	H⋯*A*	*D*⋯*A*	*D*—H⋯*A*
C42—H42*A*⋯O8^i^	0.99	2.65	3.269 (5)	121
C44—H44⋯O4	0.95	2.66	3.374 (5)	133

Symmetry code: (i) 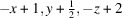 .

**Table 3:** Experimental details

Crystal data	
Chemical formula	C_17_H_18_O_3_
*M*_r_	270.31
Crystal system, space group	Monoclinic, *P*2_1_
Temperature (K)	173
*a*, *b*, *c* (Å)	16.1906 (10), 7.5752 (4), 17.7591 (9)
β (°)	95.360 (7)
*V* (Å^3^)	2168.6 (2)
*Z*	6
Radiation type	Mo *K*α
μ (mm^−1^)	0.08
Crystal size (mm)	0.30 × 0.20 × 0.05

Data collection	
Diffractometer	Rigaku R-AXIS RAPID
Absorption correction	Multi-scan (*ABSCOR*; Higashi, 1995 ▸)
*T*_min_, *T*_max_	0.533, 0.996
No. of measured, independent and observed [*I* > 2σ(*I*)] reflections	16200, 7609, 5301
*R*_int_	0.042
(sin θ/λ)_max_ (Å^−1^)	0.595

Refinement	
*R*[*F* ^2^ > 2σ(*F* ^2^)], *wR*(*F* ^2^), *S*	0.051, 0.125, 0.97
No. of reflections	7609
No. of parameters	560
No. of restraints	5
H-atom treatment	H-atom parameters constrained
Δρ_max_, Δρ_min_ (e Å^−3^)	0.16, −0.17
Absolute structure	Refined as an inversion twin
Absolute structure parameter	0.6 (14)

Computer programs: *CrystalStructure* (Rigaku, 2018 ▸), *SHELXT* (Sheldrick, 2015*a* ▸), *SHELXL* (Sheldrick, 2015*b* ▸), *DIAMOND* (Brandenburg, 1999 ▸) and *WinGX* (Farrugia, 2012 ▸).

## supplementary crystallographic information

### Crystal data

**Table:**

C_17_H_18_O_3_	*F*(000) = 864
*M**_r_* = 270.31	*D*_x_ = 1.242 Mg m^−^^3^
Monoclinic, *P*2_1_	Mo *K*α radiation, λ = 0.71075 Å
*a* = 16.1906 (10) Å	Cell parameters from 13948 reflections
*b* = 7.5752 (4) Å	θ = 2.3–27.5°
*c* = 17.7591 (9) Å	µ = 0.08 mm^−^^1^
β = 95.360 (7)°	*T* = 173 K
*V* = 2168.6 (2) Å^3^	Prism, colorless
*Z* = 6	0.30 × 0.20 × 0.05 mm

### Data collection

**Table:**

Rigaku R-AXIS RAPID diffractometer	5301 reflections with *I* > 2σ(*I*)
Detector resolution: 10.000 pixels mm^-1^	*R*_int_ = 0.042
ω scans	θ_max_ = 25.0°, θ_min_ = 2.5°
Absorption correction: multi-scan (*ABSCOR*; Higashi, 1995)	*h* = −19→19
*T*_min_ = 0.533, *T*_max_ = 0.996	*k* = −9→9
16200 measured reflections	*l* = −21→21
7609 independent reflections	

### Refinement

**Table:**

Refinement on *F*^2^	Hydrogen site location: inferred from neighbouring sites
Least-squares matrix: full	H-atom parameters constrained
*R*[*F*^2^ > 2σ(*F*^2^)] = 0.051	*w* = 1/[σ^2^(*F*_o_^2^) + (0.0667*P*)^2^] where *P* = (*F*_o_^2^ + 2*F*_c_^2^)/3
*wR*(*F*^2^) = 0.125	(Δ/σ)_max_ = 0.001
*S* = 0.97	Δρ_max_ = 0.16 e Å^−^^3^
7609 reflections	Δρ_min_ = −0.17 e Å^−^^3^
560 parameters	Absolute structure: Refined as an inversion twin
5 restraints	Absolute structure parameter: 0.6 (14)

### Special details

**Table:**

Geometry. All esds (except the esd in the dihedral angle between two l.s. planes) are estimated using the full covariance matrix. The cell esds are taken into account individually in the estimation of esds in distances, angles and torsion angles; correlations between esds in cell parameters are only used when they are defined by crystal symmetry. An approximate (isotropic) treatment of cell esds is used for estimating esds involving l.s. planes.
Refinement. Refined as a 2-component inversion twin.

### Fractional atomic coordinates and isotropic or equivalent isotropic displacement parameters (Å^2^)

**Table:**

	*x*	*y*	*z*	*U*_iso_*/*U*_eq_	Occ. (<1)
O1	0.10822 (15)	0.3014 (4)	0.12187 (14)	0.0520 (7)	
O2	0.19050 (18)	0.4249 (6)	0.47508 (17)	0.0864 (12)	
O3	0.06054 (17)	0.3271 (5)	0.47163 (14)	0.0648 (8)	
O4	0.24935 (14)	0.7272 (4)	1.05090 (15)	0.0496 (7)	
O5	0.14434 (17)	0.5821 (4)	0.70340 (16)	0.0648 (8)	
O6	0.26005 (16)	0.7442 (4)	0.69644 (15)	0.0625 (8)	
O7	0.40986 (15)	0.2922 (3)	1.18050 (15)	0.0510 (7)	
O8	0.44677 (16)	0.0780 (4)	0.83691 (16)	0.0599 (8)	
C1	0.0495 (2)	0.2152 (5)	−0.0013 (2)	0.0443 (9)	
C2	−0.0147 (2)	0.2495 (5)	−0.0573 (2)	0.0489 (10)	
H2	−0.066049	0.293542	−0.043362	0.059*	
C3	−0.0046 (2)	0.2202 (6)	−0.1331 (2)	0.0543 (11)	
H3	−0.049429	0.242504	−0.170299	0.065*	
C4	0.0702 (2)	0.1589 (6)	−0.1552 (2)	0.0497 (10)	
C5	0.1338 (2)	0.1273 (6)	−0.0996 (2)	0.0527 (10)	
H5	0.185817	0.087533	−0.113626	0.063*	
C6	0.1236 (2)	0.1522 (6)	−0.0237 (2)	0.0510 (10)	
H6	0.167989	0.125624	0.013392	0.061*	
C7	0.0823 (3)	0.1340 (7)	−0.2379 (2)	0.0659 (13)	
H7A	0.029618	0.098181	−0.265425	0.079*	
H7B	0.124086	0.042428	−0.243164	0.079*	
H7C	0.100868	0.245290	−0.258874	0.079*	
C8	0.0345 (2)	0.2349 (6)	0.0806 (2)	0.0482 (10)	
H8A	−0.012131	0.317425	0.085443	0.058*	
H8B	0.019711	0.119154	0.101448	0.058*	
C9	0.1080 (2)	0.3143 (6)	0.1992 (2)	0.0461 (9)	
C10	0.1767 (2)	0.3941 (6)	0.2365 (2)	0.0558 (11)	
H10	0.219776	0.438259	0.208893	0.067*	
C11	0.1824 (2)	0.4095 (6)	0.3143 (2)	0.0578 (11)	
H11	0.229986	0.463783	0.339851	0.069*	
C12	0.1200 (2)	0.3471 (6)	0.3559 (2)	0.0498 (10)	
C13	0.0515 (2)	0.2667 (5)	0.3178 (2)	0.0497 (10)	
H13	0.008507	0.222475	0.345492	0.060*	
C14	0.0448 (2)	0.2498 (5)	0.2392 (2)	0.0487 (10)	
H14	−0.002383	0.194827	0.213432	0.058*	
C15	0.1293 (2)	0.3687 (7)	0.4396 (2)	0.0584 (12)	
C16	0.0629 (3)	0.3580 (9)	0.5529 (2)	0.0770 (15)	
H16A	0.079550	0.481406	0.564682	0.092*	
H16B	0.103932	0.278330	0.580244	0.092*	
C17	−0.0198 (3)	0.3242 (9)	0.5765 (3)	0.0857 (16)	
H17A	−0.059461	0.407351	0.551013	0.103*	
H17B	−0.018733	0.339427	0.631386	0.103*	
H17C	−0.036552	0.203129	0.562945	0.103*	
C18	0.3073 (2)	0.8300 (5)	1.1705 (2)	0.0478 (10)	
C19	0.3626 (2)	0.7371 (6)	1.2195 (2)	0.0524 (10)	
H19	0.406274	0.672221	1.200302	0.063*	
C20	0.3545 (2)	0.7383 (6)	1.2969 (2)	0.0583 (11)	
H20	0.393475	0.675592	1.330204	0.070*	
C21	0.2907 (3)	0.8295 (6)	1.3260 (2)	0.0594 (11)	
C22	0.2347 (3)	0.9185 (6)	1.2765 (3)	0.0628 (12)	
H22	0.189626	0.979302	1.295321	0.075*	
C23	0.2433 (2)	0.9204 (6)	1.1995 (2)	0.0572 (11)	
H23	0.204792	0.984611	1.166346	0.069*	
C24	0.2827 (3)	0.8334 (8)	1.4110 (2)	0.0822 (16)	
H24A	0.336608	0.806262	1.438416	0.099*	
H24B	0.241827	0.745422	1.423610	0.099*	
H24C	0.264674	0.951011	1.425594	0.099*	
C25	0.3159 (2)	0.8310 (6)	1.0874 (2)	0.0523 (10)	
H25A	0.312822	0.953494	1.067897	0.063*	
H25B	0.370048	0.780204	1.077218	0.063*	
C26	0.2446 (2)	0.7150 (5)	0.9736 (2)	0.0432 (9)	
C27	0.1790 (2)	0.6128 (5)	0.9411 (2)	0.0470 (10)	
H27	0.142358	0.556082	0.972200	0.056*	
C28	0.1676 (2)	0.5945 (5)	0.8635 (2)	0.0489 (10)	
H28	0.122810	0.525418	0.841335	0.059*	
C29	0.2212 (2)	0.6763 (5)	0.8173 (2)	0.0457 (9)	
C30	0.2876 (2)	0.7729 (5)	0.8507 (2)	0.0476 (10)	
H30	0.325151	0.826994	0.819724	0.057*	
C31	0.3002 (2)	0.7918 (5)	0.9286 (2)	0.0476 (10)	
H31	0.346440	0.856677	0.950938	0.057*	
C32	0.2039 (2)	0.6608 (6)	0.7346 (2)	0.0517 (10)	
C33	0.2436 (3)	0.7472 (8)	0.6140 (2)	0.0743 (15)	
H33A	0.220428	0.632046	0.596184	0.089*	
H33B	0.296151	0.766405	0.590866	0.089*	
C34	0.1840 (3)	0.8902 (10)	0.5902 (3)	0.0973 (19)	
H34A	0.174983	0.892892	0.534905	0.117*	
H34B	0.206633	1.003831	0.608704	0.117*	
H34C	0.131214	0.868111	0.611367	0.117*	
C35	0.4655 (2)	0.2889 (5)	1.3101 (2)	0.0491 (10)	
C36	0.5264 (2)	0.3902 (6)	1.3491 (2)	0.0564 (11)	
H36	0.574469	0.421203	1.325178	0.068*	
C37	0.5190 (3)	0.4475 (6)	1.4222 (3)	0.0598 (11)	
H37	0.562457	0.514344	1.448032	0.072*	
C38	0.4486 (3)	0.4081 (6)	1.4581 (2)	0.0572 (11)	
C39	0.3864 (3)	0.3103 (6)	1.4183 (2)	0.0602 (11)	
H39	0.337265	0.283573	1.441409	0.072*	
C40	0.3948 (2)	0.2508 (6)	1.3455 (2)	0.0579 (11)	
H40	0.351576	0.183268	1.319594	0.070*	
C41	0.4391 (3)	0.4632 (7)	1.5379 (2)	0.0780 (14)	
H41A	0.380484	0.456245	1.547340	0.094*	
H41B	0.458706	0.584850	1.545643	0.094*	
H41C	0.471799	0.384621	1.572923	0.094*	
C42	0.4754 (2)	0.2209 (6)	1.2318 (2)	0.0536 (10)	
H42A	0.529913	0.257174	1.216028	0.064*	
H42B	0.472692	0.090338	1.231359	0.064*	
C43	0.4135 (2)	0.2591 (5)	1.1059 (2)	0.0462 (10)	
C44	0.3508 (2)	0.3376 (5)	1.0580 (2)	0.0493 (10)	
H44	0.308706	0.404410	1.078743	0.059*	
C45	0.3497 (2)	0.3187 (5)	0.9804 (2)	0.0531 (10)	
H45	0.307418	0.374312	0.948115	0.064*	
C46	0.4106 (2)	0.2179 (5)	0.9493 (2)	0.0476 (10)	
C47	0.4712 (2)	0.1361 (5)	0.9978 (2)	0.0482 (10)	
H47	0.511533	0.064016	0.977267	0.058*	
C48	0.4739 (2)	0.1577 (5)	1.0761 (2)	0.0464 (9)	
H48	0.516601	0.103709	1.108483	0.056*	
C49	0.4124 (2)	0.1963 (6)	0.8672 (3)	0.0584 (11)	
O9	0.3807 (3)	0.3384 (6)	0.8254 (2)	0.0544 (16)	0.735 (9)
C50	0.3807 (5)	0.3379 (13)	0.7433 (6)	0.064 (2)	0.735 (9)
H50A	0.432423	0.282505	0.729022	0.077*	0.735 (9)
H50B	0.378868	0.460869	0.724260	0.077*	0.735 (9)
C51	0.3068 (4)	0.2372 (9)	0.7079 (4)	0.067 (2)	0.735 (9)
H51A	0.307393	0.237694	0.652755	0.101*	0.735 (9)
H51B	0.309176	0.115223	0.726315	0.101*	0.735 (9)
H51C	0.255724	0.293246	0.721562	0.101*	0.735 (9)
O9B	0.3346 (8)	0.2613 (17)	0.8386 (6)	0.056 (4)*	0.265 (9)
C50B	0.3182 (13)	0.228 (3)	0.7576 (11)	0.078 (6)*	0.265 (9)
H50C	0.260686	0.263008	0.740216	0.093*	0.265 (9)
H50D	0.324382	0.100218	0.747293	0.093*	0.265 (9)
C51B	0.377 (2)	0.330 (5)	0.7170 (19)	0.081 (12)*	0.265 (9)
H51D	0.366615	0.307989	0.662536	0.122*	0.265 (9)
H51E	0.370481	0.455896	0.727113	0.122*	0.265 (9)
H51F	0.433994	0.293573	0.734169	0.122*	0.265 (9)

### Atomic displacement parameters (Å^2^)

**Table:**

	*U*^11^	*U*^22^	*U*^33^	*U*^12^	*U*^13^	*U*^23^
O1	0.0389 (14)	0.0603 (19)	0.0572 (17)	−0.0066 (13)	0.0069 (11)	−0.0051 (15)
O2	0.0544 (19)	0.136 (4)	0.069 (2)	−0.016 (2)	0.0050 (15)	−0.028 (2)
O3	0.0547 (17)	0.085 (2)	0.0546 (17)	−0.0051 (17)	0.0072 (13)	−0.0107 (17)
O4	0.0407 (14)	0.0462 (16)	0.0617 (18)	−0.0072 (13)	0.0043 (12)	0.0012 (14)
O5	0.0518 (17)	0.072 (2)	0.0698 (19)	−0.0104 (17)	0.0028 (14)	−0.0147 (17)
O6	0.0508 (16)	0.079 (2)	0.0583 (18)	−0.0110 (16)	0.0111 (13)	−0.0097 (16)
O7	0.0411 (14)	0.0503 (17)	0.0619 (18)	0.0051 (13)	0.0060 (12)	0.0001 (15)
O8	0.0538 (16)	0.0573 (19)	0.0695 (19)	0.0051 (15)	0.0096 (13)	−0.0054 (16)
C1	0.039 (2)	0.036 (2)	0.058 (2)	−0.0038 (18)	0.0085 (17)	−0.0015 (19)
C2	0.040 (2)	0.045 (2)	0.063 (3)	−0.0003 (18)	0.0106 (18)	0.002 (2)
C3	0.046 (2)	0.056 (3)	0.060 (3)	−0.007 (2)	−0.0004 (18)	0.007 (2)
C4	0.051 (2)	0.043 (2)	0.056 (2)	−0.0052 (19)	0.0115 (19)	0.002 (2)
C5	0.042 (2)	0.052 (3)	0.066 (3)	0.0035 (19)	0.0130 (19)	−0.002 (2)
C6	0.041 (2)	0.052 (2)	0.061 (3)	0.0001 (19)	0.0054 (18)	0.003 (2)
C7	0.078 (3)	0.063 (3)	0.058 (3)	−0.016 (3)	0.014 (2)	−0.004 (2)
C8	0.040 (2)	0.050 (2)	0.055 (2)	−0.0056 (19)	0.0085 (17)	−0.002 (2)
C9	0.040 (2)	0.047 (2)	0.052 (2)	0.0026 (19)	0.0081 (16)	−0.004 (2)
C10	0.044 (2)	0.060 (3)	0.066 (3)	−0.008 (2)	0.0137 (19)	−0.008 (2)
C11	0.044 (2)	0.059 (3)	0.071 (3)	−0.009 (2)	0.0030 (19)	−0.012 (2)
C12	0.041 (2)	0.052 (3)	0.056 (2)	0.003 (2)	0.0038 (17)	−0.006 (2)
C13	0.040 (2)	0.050 (3)	0.059 (3)	−0.0032 (19)	0.0079 (17)	−0.003 (2)
C14	0.037 (2)	0.052 (3)	0.056 (3)	−0.0034 (19)	0.0025 (17)	−0.008 (2)
C15	0.045 (2)	0.069 (3)	0.061 (3)	0.003 (2)	0.003 (2)	−0.012 (2)
C16	0.072 (3)	0.112 (4)	0.048 (3)	0.004 (3)	0.005 (2)	−0.010 (3)
C17	0.086 (4)	0.111 (5)	0.061 (3)	−0.012 (3)	0.012 (2)	−0.002 (3)
C18	0.041 (2)	0.039 (2)	0.063 (3)	−0.0033 (19)	0.0035 (18)	0.001 (2)
C19	0.042 (2)	0.049 (3)	0.066 (3)	0.004 (2)	0.0088 (18)	0.000 (2)
C20	0.049 (2)	0.058 (3)	0.067 (3)	0.000 (2)	−0.0007 (19)	0.011 (2)
C21	0.063 (3)	0.053 (3)	0.063 (3)	−0.011 (2)	0.011 (2)	−0.001 (2)
C22	0.055 (3)	0.058 (3)	0.077 (3)	0.008 (2)	0.014 (2)	−0.009 (3)
C23	0.051 (2)	0.053 (3)	0.067 (3)	0.006 (2)	0.001 (2)	−0.004 (2)
C24	0.093 (4)	0.088 (4)	0.067 (3)	−0.011 (3)	0.019 (3)	−0.003 (3)
C25	0.040 (2)	0.055 (3)	0.062 (3)	−0.007 (2)	0.0046 (17)	−0.007 (2)
C26	0.0363 (19)	0.038 (2)	0.056 (2)	0.0034 (18)	0.0050 (16)	−0.0020 (19)
C27	0.033 (2)	0.040 (2)	0.069 (3)	0.0010 (17)	0.0104 (18)	0.000 (2)
C28	0.0320 (19)	0.041 (2)	0.073 (3)	0.0008 (18)	0.0020 (18)	−0.005 (2)
C29	0.0339 (19)	0.043 (2)	0.060 (3)	0.0045 (17)	0.0030 (17)	−0.005 (2)
C30	0.039 (2)	0.043 (2)	0.062 (3)	−0.0006 (18)	0.0115 (17)	−0.003 (2)
C31	0.036 (2)	0.044 (2)	0.062 (3)	−0.0045 (17)	0.0033 (17)	−0.004 (2)
C32	0.039 (2)	0.051 (3)	0.065 (3)	0.000 (2)	0.0068 (19)	−0.013 (2)
C33	0.064 (3)	0.105 (4)	0.056 (3)	−0.018 (3)	0.018 (2)	−0.022 (3)
C34	0.084 (4)	0.141 (6)	0.067 (3)	0.000 (4)	0.006 (3)	0.009 (4)
C35	0.041 (2)	0.043 (2)	0.064 (3)	0.0045 (18)	0.0064 (18)	0.006 (2)
C36	0.043 (2)	0.050 (3)	0.076 (3)	−0.003 (2)	0.006 (2)	0.007 (2)
C37	0.051 (2)	0.050 (3)	0.077 (3)	−0.007 (2)	−0.003 (2)	0.000 (2)
C38	0.060 (3)	0.048 (3)	0.063 (3)	0.000 (2)	0.001 (2)	0.000 (2)
C39	0.050 (2)	0.063 (3)	0.070 (3)	−0.005 (2)	0.018 (2)	0.000 (2)
C40	0.042 (2)	0.057 (3)	0.075 (3)	−0.009 (2)	0.0052 (19)	−0.001 (2)
C41	0.093 (4)	0.068 (3)	0.073 (3)	0.001 (3)	0.009 (3)	−0.008 (3)
C42	0.039 (2)	0.057 (3)	0.065 (3)	0.004 (2)	0.0051 (18)	0.006 (2)
C43	0.0341 (19)	0.040 (2)	0.066 (3)	−0.0026 (18)	0.0103 (17)	−0.003 (2)
C44	0.036 (2)	0.042 (2)	0.070 (3)	0.0046 (18)	0.0045 (18)	−0.005 (2)
C45	0.040 (2)	0.042 (2)	0.075 (3)	0.0058 (19)	−0.0065 (18)	−0.006 (2)
C46	0.039 (2)	0.041 (2)	0.061 (3)	−0.0001 (19)	−0.0004 (17)	−0.006 (2)
C47	0.037 (2)	0.036 (2)	0.073 (3)	0.0008 (18)	0.0074 (18)	−0.002 (2)
C48	0.0339 (19)	0.042 (2)	0.063 (3)	−0.0001 (18)	0.0018 (17)	0.003 (2)
C49	0.049 (2)	0.052 (3)	0.072 (3)	0.009 (2)	−0.005 (2)	−0.011 (2)
O9	0.061 (3)	0.046 (3)	0.056 (3)	0.008 (2)	0.0020 (18)	0.001 (2)
C50	0.058 (4)	0.062 (5)	0.074 (7)	−0.006 (3)	0.012 (4)	−0.005 (5)
C51	0.052 (4)	0.064 (4)	0.084 (6)	−0.003 (3)	−0.002 (3)	0.000 (4)

### Geometric parameters (Å, º)

**Table:**

O1—C9	1.377 (4)	C25—H25B	0.9900
O1—C8	1.432 (4)	C26—C31	1.387 (5)
O2—C15	1.201 (5)	C26—C27	1.395 (5)
O3—C15	1.335 (5)	C27—C28	1.380 (5)
O3—C16	1.459 (4)	C27—H27	0.9500
O4—C26	1.371 (4)	C28—C29	1.393 (5)
O4—C25	1.438 (4)	C28—H28	0.9500
O5—C32	1.222 (4)	C29—C30	1.387 (5)
O6—C32	1.341 (5)	C29—C32	1.475 (5)
O6—C33	1.463 (5)	C30—C31	1.387 (5)
O7—C43	1.355 (4)	C30—H30	0.9500
O7—C42	1.438 (4)	C31—H31	0.9500
O8—C49	1.207 (5)	C33—C34	1.485 (8)
C1—C6	1.383 (5)	C33—H33A	0.9900
C1—C2	1.393 (5)	C33—H33B	0.9900
C1—C8	1.505 (5)	C34—H34A	0.9800
C2—C3	1.390 (5)	C34—H34B	0.9800
C2—H2	0.9500	C34—H34C	0.9800
C3—C4	1.387 (5)	C35—C36	1.384 (5)
C3—H3	0.9500	C35—C40	1.387 (5)
C4—C5	1.379 (5)	C35—C42	1.505 (5)
C4—C7	1.512 (5)	C36—C37	1.384 (6)
C5—C6	1.386 (5)	C36—H36	0.9500
C5—H5	0.9500	C37—C38	1.389 (6)
C6—H6	0.9500	C37—H37	0.9500
C7—H7A	0.9800	C38—C39	1.389 (6)
C7—H7B	0.9800	C38—C41	1.500 (6)
C7—H7C	0.9800	C39—C40	1.388 (6)
C8—H8A	0.9900	C39—H39	0.9500
C8—H8B	0.9900	C40—H40	0.9500
C9—C10	1.380 (5)	C41—H41A	0.9800
C9—C14	1.388 (5)	C41—H41B	0.9800
C10—C11	1.380 (5)	C41—H41C	0.9800
C10—H10	0.9500	C42—H42A	0.9900
C11—C12	1.389 (5)	C42—H42B	0.9900
C11—H11	0.9500	C43—C48	1.387 (5)
C12—C13	1.386 (5)	C43—C44	1.395 (5)
C12—C15	1.488 (5)	C44—C45	1.384 (5)
C13—C14	1.395 (5)	C44—H44	0.9500
C13—H13	0.9500	C45—C46	1.401 (5)
C14—H14	0.9500	C45—H45	0.9500
C16—C17	1.463 (6)	C46—C47	1.389 (5)
C16—H16A	0.9900	C46—C49	1.471 (6)
C16—H16B	0.9900	C47—C48	1.397 (5)
C17—H17A	0.9800	C47—H47	0.9500
C17—H17B	0.9800	C48—H48	0.9500
C17—H17C	0.9800	C49—O9	1.379 (6)
C18—C23	1.381 (5)	C49—O9B	1.403 (12)
C18—C19	1.382 (5)	O9—C50	1.458 (11)
C18—C25	1.495 (5)	C50—C51	1.507 (10)
C19—C20	1.394 (5)	C50—H50A	0.9900
C19—H19	0.9500	C50—H50B	0.9900
C20—C21	1.383 (6)	C51—H51A	0.9800
C20—H20	0.9500	C51—H51B	0.9800
C21—C22	1.379 (6)	C51—H51C	0.9800
C21—C24	1.527 (5)	O9B—C50B	1.459 (19)
C22—C23	1.388 (5)	C50B—C51B	1.47 (2)
C22—H22	0.9500	C50B—H50C	0.9900
C23—H23	0.9500	C50B—H50D	0.9900
C24—H24A	0.9800	C51B—H51D	0.9800
C24—H24B	0.9800	C51B—H51E	0.9800
C24—H24C	0.9800	C51B—H51F	0.9800
C25—H25A	0.9900		
			
C9—O1—C8	117.1 (3)	C27—C28—H28	119.6
C15—O3—C16	115.9 (3)	C29—C28—H28	119.6
C26—O4—C25	117.3 (3)	C30—C29—C28	118.9 (4)
C32—O6—C33	116.3 (3)	C30—C29—C32	122.4 (3)
C43—O7—C42	117.0 (3)	C28—C29—C32	118.7 (3)
C6—C1—C2	117.9 (4)	C31—C30—C29	121.1 (3)
C6—C1—C8	122.3 (3)	C31—C30—H30	119.4
C2—C1—C8	119.6 (3)	C29—C30—H30	119.4
C3—C2—C1	120.9 (3)	C26—C31—C30	119.2 (4)
C3—C2—H2	119.5	C26—C31—H31	120.4
C1—C2—H2	119.5	C30—C31—H31	120.4
C4—C3—C2	120.8 (4)	O5—C32—O6	123.0 (4)
C4—C3—H3	119.6	O5—C32—C29	124.0 (4)
C2—C3—H3	119.6	O6—C32—C29	113.0 (3)
C5—C4—C3	117.9 (4)	O6—C33—C34	110.4 (4)
C5—C4—C7	121.3 (4)	O6—C33—H33A	109.6
C3—C4—C7	120.8 (4)	C34—C33—H33A	109.6
C4—C5—C6	121.6 (4)	O6—C33—H33B	109.6
C4—C5—H5	119.2	C34—C33—H33B	109.6
C6—C5—H5	119.2	H33A—C33—H33B	108.1
C1—C6—C5	120.7 (4)	C33—C34—H34A	109.5
C1—C6—H6	119.6	C33—C34—H34B	109.5
C5—C6—H6	119.6	H34A—C34—H34B	109.5
C4—C7—H7A	109.5	C33—C34—H34C	109.5
C4—C7—H7B	109.5	H34A—C34—H34C	109.5
H7A—C7—H7B	109.5	H34B—C34—H34C	109.5
C4—C7—H7C	109.5	C36—C35—C40	118.0 (4)
H7A—C7—H7C	109.5	C36—C35—C42	121.3 (3)
H7B—C7—H7C	109.5	C40—C35—C42	120.7 (4)
O1—C8—C1	109.1 (3)	C35—C36—C37	121.5 (4)
O1—C8—H8A	109.9	C35—C36—H36	119.2
C1—C8—H8A	109.9	C37—C36—H36	119.2
O1—C8—H8B	109.9	C36—C37—C38	120.7 (4)
C1—C8—H8B	109.9	C36—C37—H37	119.7
H8A—C8—H8B	108.3	C38—C37—H37	119.7
O1—C9—C10	115.7 (3)	C37—C38—C39	118.0 (4)
O1—C9—C14	123.8 (3)	C37—C38—C41	122.4 (4)
C10—C9—C14	120.5 (4)	C39—C38—C41	119.6 (4)
C9—C10—C11	119.6 (4)	C40—C39—C38	121.1 (4)
C9—C10—H10	120.2	C40—C39—H39	119.4
C11—C10—H10	120.2	C38—C39—H39	119.4
C10—C11—C12	121.3 (4)	C35—C40—C39	120.8 (4)
C10—C11—H11	119.3	C35—C40—H40	119.6
C12—C11—H11	119.3	C39—C40—H40	119.6
C13—C12—C11	118.6 (4)	C38—C41—H41A	109.5
C13—C12—C15	122.5 (4)	C38—C41—H41B	109.5
C11—C12—C15	118.9 (4)	H41A—C41—H41B	109.5
C12—C13—C14	120.8 (4)	C38—C41—H41C	109.5
C12—C13—H13	119.6	H41A—C41—H41C	109.5
C14—C13—H13	119.6	H41B—C41—H41C	109.5
C9—C14—C13	119.2 (3)	O7—C42—C35	108.7 (3)
C9—C14—H14	120.4	O7—C42—H42A	109.9
C13—C14—H14	120.4	C35—C42—H42A	109.9
O2—C15—O3	122.7 (4)	O7—C42—H42B	109.9
O2—C15—C12	124.3 (4)	C35—C42—H42B	109.9
O3—C15—C12	112.9 (3)	H42A—C42—H42B	108.3
O3—C16—C17	108.4 (4)	O7—C43—C48	124.9 (4)
O3—C16—H16A	110.0	O7—C43—C44	114.9 (3)
C17—C16—H16A	110.0	C48—C43—C44	120.2 (4)
O3—C16—H16B	110.0	C45—C44—C43	120.2 (4)
C17—C16—H16B	110.0	C45—C44—H44	119.9
H16A—C16—H16B	108.4	C43—C44—H44	119.9
C16—C17—H17A	109.5	C44—C45—C46	120.4 (4)
C16—C17—H17B	109.5	C44—C45—H45	119.8
H17A—C17—H17B	109.5	C46—C45—H45	119.8
C16—C17—H17C	109.5	C47—C46—C45	118.7 (4)
H17A—C17—H17C	109.5	C47—C46—C49	119.1 (4)
H17B—C17—H17C	109.5	C45—C46—C49	122.2 (4)
C23—C18—C19	118.9 (4)	C46—C47—C48	121.3 (3)
C23—C18—C25	120.5 (4)	C46—C47—H47	119.3
C19—C18—C25	120.6 (3)	C48—C47—H47	119.3
C18—C19—C20	120.2 (4)	C43—C48—C47	119.1 (4)
C18—C19—H19	119.9	C43—C48—H48	120.4
C20—C19—H19	119.9	C47—C48—H48	120.4
C21—C20—C19	120.9 (4)	O8—C49—O9	120.3 (4)
C21—C20—H20	119.5	O8—C49—O9B	122.3 (6)
C19—C20—H20	119.5	O8—C49—C46	125.2 (4)
C22—C21—C20	118.4 (4)	O9—C49—C46	113.9 (4)
C22—C21—C24	120.7 (4)	O9B—C49—C46	102.7 (5)
C20—C21—C24	120.9 (4)	C49—O9—C50	120.1 (5)
C21—C22—C23	120.9 (4)	O9—C50—C51	110.1 (7)
C21—C22—H22	119.5	O9—C50—H50A	109.6
C23—C22—H22	119.5	C51—C50—H50A	109.6
C18—C23—C22	120.7 (4)	O9—C50—H50B	109.6
C18—C23—H23	119.7	C51—C50—H50B	109.6
C22—C23—H23	119.7	H50A—C50—H50B	108.2
C21—C24—H24A	109.5	C50—C51—H51A	109.5
C21—C24—H24B	109.5	C50—C51—H51B	109.5
H24A—C24—H24B	109.5	H51A—C51—H51B	109.5
C21—C24—H24C	109.5	C50—C51—H51C	109.5
H24A—C24—H24C	109.5	H51A—C51—H51C	109.5
H24B—C24—H24C	109.5	H51B—C51—H51C	109.5
O4—C25—C18	107.9 (3)	C49—O9B—C50B	111.7 (11)
O4—C25—H25A	110.1	O9B—C50B—C51B	109 (2)
C18—C25—H25A	110.1	O9B—C50B—H50C	109.9
O4—C25—H25B	110.1	C51B—C50B—H50C	109.9
C18—C25—H25B	110.1	O9B—C50B—H50D	109.9
H25A—C25—H25B	108.4	C51B—C50B—H50D	109.9
O4—C26—C31	124.7 (3)	H50C—C50B—H50D	108.3
O4—C26—C27	114.9 (3)	C50B—C51B—H51D	109.5
C31—C26—C27	120.3 (4)	C50B—C51B—H51E	109.5
C28—C27—C26	119.6 (3)	H51D—C51B—H51E	109.5
C28—C27—H27	120.2	C50B—C51B—H51F	109.5
C26—C27—H27	120.2	H51D—C51B—H51F	109.5
C27—C28—C29	120.7 (4)	H51E—C51B—H51F	109.5
			
C6—C1—C2—C3	0.4 (6)	C27—C28—C29—C32	−176.7 (3)
C8—C1—C2—C3	−175.3 (4)	C28—C29—C30—C31	−1.3 (5)
C1—C2—C3—C4	−1.1 (6)	C32—C29—C30—C31	177.0 (4)
C2—C3—C4—C5	0.3 (6)	O4—C26—C31—C30	−178.7 (3)
C2—C3—C4—C7	−177.6 (4)	C27—C26—C31—C30	3.1 (6)
C3—C4—C5—C6	1.2 (6)	C29—C30—C31—C26	−1.0 (6)
C7—C4—C5—C6	179.1 (4)	C33—O6—C32—O5	4.1 (6)
C2—C1—C6—C5	1.2 (6)	C33—O6—C32—C29	−175.2 (4)
C8—C1—C6—C5	176.7 (4)	C30—C29—C32—O5	−177.3 (4)
C4—C5—C6—C1	−2.0 (6)	C28—C29—C32—O5	1.0 (6)
C9—O1—C8—C1	−174.9 (3)	C30—C29—C32—O6	2.0 (5)
C6—C1—C8—O1	40.1 (5)	C28—C29—C32—O6	−179.6 (4)
C2—C1—C8—O1	−144.3 (4)	C32—O6—C33—C34	81.8 (5)
C8—O1—C9—C10	−174.1 (4)	C40—C35—C36—C37	2.2 (6)
C8—O1—C9—C14	7.1 (6)	C42—C35—C36—C37	−177.5 (4)
O1—C9—C10—C11	−178.7 (4)	C35—C36—C37—C38	−1.6 (7)
C14—C9—C10—C11	0.1 (6)	C36—C37—C38—C39	−0.1 (7)
C9—C10—C11—C12	−0.4 (7)	C36—C37—C38—C41	178.3 (4)
C10—C11—C12—C13	0.6 (7)	C37—C38—C39—C40	1.1 (7)
C10—C11—C12—C15	−179.5 (4)	C41—C38—C39—C40	−177.3 (4)
C11—C12—C13—C14	−0.5 (6)	C36—C35—C40—C39	−1.1 (6)
C15—C12—C13—C14	179.6 (4)	C42—C35—C40—C39	178.6 (4)
O1—C9—C14—C13	178.8 (4)	C38—C39—C40—C35	−0.5 (7)
C10—C9—C14—C13	0.0 (6)	C43—O7—C42—C35	173.7 (3)
C12—C13—C14—C9	0.2 (6)	C36—C35—C42—O7	−118.9 (4)
C16—O3—C15—O2	1.5 (7)	C40—C35—C42—O7	61.4 (5)
C16—O3—C15—C12	−175.5 (4)	C42—O7—C43—C48	2.1 (5)
C13—C12—C15—O2	173.9 (5)	C42—O7—C43—C44	−177.2 (3)
C11—C12—C15—O2	−6.0 (7)	O7—C43—C44—C45	177.6 (3)
C13—C12—C15—O3	−9.3 (6)	C48—C43—C44—C45	−1.6 (6)
C11—C12—C15—O3	170.9 (4)	C43—C44—C45—C46	1.2 (6)
C15—O3—C16—C17	173.7 (5)	C44—C45—C46—C47	0.8 (6)
C23—C18—C19—C20	−1.3 (6)	C44—C45—C46—C49	−179.1 (4)
C25—C18—C19—C20	179.4 (4)	C45—C46—C47—C48	−2.2 (6)
C18—C19—C20—C21	1.0 (7)	C49—C46—C47—C48	177.6 (4)
C19—C20—C21—C22	0.4 (7)	O7—C43—C48—C47	−179.0 (3)
C19—C20—C21—C24	−178.9 (4)	C44—C43—C48—C47	0.2 (5)
C20—C21—C22—C23	−1.6 (7)	C46—C47—C48—C43	1.8 (5)
C24—C21—C22—C23	177.7 (4)	C47—C46—C49—O8	21.5 (6)
C19—C18—C23—C22	0.1 (6)	C45—C46—C49—O8	−158.6 (4)
C25—C18—C23—C22	179.4 (4)	C47—C46—C49—O9	−149.8 (4)
C21—C22—C23—C18	1.4 (7)	C45—C46—C49—O9	30.1 (6)
C26—O4—C25—C18	178.3 (3)	C47—C46—C49—O9B	167.6 (7)
C23—C18—C25—O4	−70.6 (5)	C45—C46—C49—O9B	−12.5 (8)
C19—C18—C25—O4	108.7 (4)	O8—C49—O9—C50	6.4 (8)
C25—O4—C26—C31	1.6 (5)	C46—C49—O9—C50	178.2 (5)
C25—O4—C26—C27	180.0 (3)	C49—O9—C50—C51	82.8 (8)
O4—C26—C27—C28	178.8 (3)	O8—C49—O9B—C50B	−24.0 (16)
C31—C26—C27—C28	−2.7 (6)	C46—C49—O9B—C50B	−171.4 (12)
C26—C27—C28—C29	0.3 (6)	C49—O9B—C50B—C51B	−66 (2)
C27—C28—C29—C30	1.7 (6)		

### Hydrogen-bond geometry (Å, º)

**Table:**

*D*—H···*A*	*D*—H	H···*A*	*D*···*A*	*D*—H···*A*
C42—H42*A*···O8^i^	0.99	2.65	3.269 (5)	121
C44—H44···O4	0.95	2.66	3.374 (5)	133

Symmetry code: (i) −*x*+1, *y*+1/2, −*z*+2.
